# The TALKS study to improve communication, logistical, and financial barriers to live donor kidney transplantation in African Americans: protocol of a randomized clinical trial

**DOI:** 10.1186/s12882-015-0153-y

**Published:** 2015-10-09

**Authors:** Tara S. Strigo, Patti L. Ephraim, Iris Pounds, Felicia Hill-Briggs, Linda Darrell, Matthew Ellis, Debra Sudan, Hamid Rabb, Dorry Segev, Nae-Yuh Wang, Mary Kaiser, Margaret Falkovic, Jill F. Lebov, L. Ebony Boulware

**Affiliations:** Division of General Internal Medicine, Duke University School of Medicine, Durham, NC 27701 USA; Department of Epidemiology, Johns Hopkins Bloomberg School of Public Health and Welch Center for Prevention, Epidemiology and Clinical Research, Johns Hopkins Medical Institutions, Baltimore, MD USA; Welch Center for Prevention, Epidemiology and Clinical Research, Johns Hopkins Medical Institution and Division of General Internal Medicine, Johns Hopkins School of Medicine, Baltimore, MD USA; Department of Social Work, Morgan State University, Baltimore, MD USA; Division of Nephrology, Duke University School of Medicine, Durham, NC USA; Division of Transplantation, Duke University School of Medicine, Durham, NC USA; Division of Nephrology, Johns Hopkins School of Medicine, Baltimore, MD USA; Division of Transplantation, Johns Hopkins School of Medicine, Baltimore, MD USA

**Keywords:** Live donor kidney transplant, Live kidney donation, African Americans, Disparities, Randomized clinical trial

## Abstract

**Background:**

Live donor kidney transplantation (LDKT), an optimal therapy for many patients with end-stage kidney disease, is underutilized, particularly by African Americans. Potential recipient difficulties initiating and sustaining conversations about LDKT, identifying willing and medically eligible donors, and potential donors’ logistical and financial hurdles have been cited as potential contributors to race disparities in LDKT. Few interventions specifically targeting these factors have been tested.

**Methods/Design:**

We report the protocol of the Talking about Living Kidney Donation Support (TALKS) study, a study designed to evaluate the effectiveness of behavioral, educational and financial assistance interventions to improve access to LDKT among African Americans on the deceased donor kidney transplant recipient waiting list. We adapted a previously tested educational and social worker intervention shown to improve consideration and pursuit of LDKT among patients and their family members for its use among patients on the kidney transplant waiting list. We also developed a financial assistance intervention to help potential donors overcome logistical and financial challenges they might face during the pursuit of live kidney donation. We will evaluate the effectiveness of these interventions by conducting a randomized controlled trial in which patients on the deceased donor waiting list receive 1) usual care while on the transplant waiting list, 2) the educational and social worker intervention, or 3) the educational and social worker intervention plus the option of participating in the financial assistance program. The primary outcome of the randomized controlled trial will measure potential recipients’ live kidney donor activation (a composite rate of live donor inquiries, completed new live donor evaluations, or live kidney donation) at 1 year.

**Discussion:**

The TALKS study will rigorously assess the effectiveness of promising interventions to reduce race disparities in LDKT.

**Trial registration:**

NCT02369354.

## Background

Live donor kidney transplantation (LDKT) represents an optimal therapy for many patients with end-stage kidney disease, and it is associated with substantially improved quality of life and survival compared to dialysis [1–3]. However, African Americans have been persistently and substantially less likely to receive LDKT when compared to Whites [4]. Numerous factors ranging from recipient difficulties initiating and completing transplant evaluations [5–7] to difficulties identifying donors [8, 9], and donor difficulties completing medical evaluations have been cited as contributors to race disparities in LDKT [10]. To date, however, few interventions specifically targeting factors that could hinder African Americans from obtaining LDKT have been tested.

The process of obtaining LDKT requires that potential recipients and donors are able to overcome several possible communication and logistical challenges, some of which may affect African Americans disproportionately and contribute to their lower rates of LDKT compared to Whites. For instance, as a critical first step in the process, patients with kidney disease, their families, and their physicians must engage in discussions about live donor kidney transplantation. Patient-physician discussions about LDKT are needed to establish whether LDKT is a viable and/or desirable treatment option for potential recipients, and discussions are needed to ensure that potential recipients understand the risks and benefits of LDKT. Family-physician discussions are also important in helping family members (who not only help potential recipients make informed treatment decisions but who also could serve as potential donors) understand the process of LDKT and associated risks and benefits. Discussions occurring within families about LDKT help families establish whether it is possible to identify willing and medically eligible live donors, and they help families discuss numerous considerations, including the potential psychological, physical, and financial strains of LDKT on family health and priorities. Studies have shown that even when African American patients want to obtain LDKT, they engage in discussions about LDKT with their physicians and their family members at suboptimal rates [11]. African Americans are also less likely than Whites to report they know about transplant prior to initiating dialysis [12, 13], and they cite numerous barriers to discussing LDKT, including their discomfort with initiating discussions, concerns about burdening family members, and their uncertainty regarding the appropriate timing for initiating discussions [14]. Once discussions have occurred, patients and their potential donors must consider numerous additional logistical and financial demands placed on potential donors during the LDKT process. For instance, potential donors may require childcare or travel to transplant centers during the evaluation process, and they may also require unpaid time away from work. Evidence suggests African Americans may be more sensitive than Whites to logistical and financial barriers to LDKT [15–17].

African Americans who are on the deceased donor kidney transplant waiting list have already overcome many barriers to seeking kidney transplants [5, 18]. However, communication, logistical and financial barriers may prevent them from pursuing LDKT while waiting for a deceased donor kidney. Evidence suggests interventions employing behavioral support personnel such as transplant social workers to help patients consider LDKT, discuss LDKT with their physicians and families, and overcome logistical barriers to considering or pursuing LDKT could facilitate African Americans’ consideration of LDKT [14, 19, 20]. Donor financial assistance programs such as the National Living Donor Assistance Center program (NLDAC) (https://www.livingdonorassistance.org/default.aspx), [21] could also help African Americans overcome financial barriers to LDKT. However, studies have not been performed to determine whether these interventions can be employed to improve African Americans’ access to LDKT.

We describe the protocol for a randomized controlled trial designed to study the effectiveness of behavioral, educational and financial assistance interventions to improve access to LDKT among African Americans on the deceased donor kidney transplant waiting list.

## Methods/ Design

### Overview

The Talking about Living Kidney Donation Support (TALKS) study is a randomized controlled trial designed to test the incremental effectiveness of (1) a culturally sensitive educational and behavioral social worker intervention and (2) a live donor financial assistance intervention to improve potential kidney recipient activation (i.e., discussions with physicians and family about LDKT) and live kidney donation among African American patients on the deceased donor kidney transplant waiting list at Duke University Medical Center. We adapted the previously developed Talking About Live Kidney Donation (TALK) Social Worker protocol [22] for use among transplant patients on the deceased donor kidney waiting list and developed a live donor financial assistance program. These interventions were designed to overcome communication, logistical, and financial barriers to LDKT (Table 1). The protocol was approved by the Duke University School of Medicine Institutional Review Board.

**Table 1:** Proposed mechanisms through which interventions lead to greater pursuit of LDKT and live kidney donation among African Americans on the deceased donor kidney transplant waiting list

Barrier to LDKT	Proposed mechanisms
	Educational and social worker support
Knowledge barriers	• Educational video and booklet introduce patients and their families to LDKT
	• Social worker refers patient to health care professionals able to discuss risks with patient and potential donors
Interpersonal difficulties initiating and sustaining LDKT discussions with family, health care team	• Educational video and booklet encourage LDKT discussions
	• Social worker encourages LDKT discussions, help patients overcome self-identified barriers to LDKT discussions
Logistical and financial barriers	• Social worker provides patients and families with information on existing financial resources for recipient and potential donor
	• Social worker offers financial assistance Intervention to assist with child care or uncovered donor expenses related to donor evaluation, donation, and donor recovery^a^

^a^Those randomly assigned to financial assistance intervention only

#### TALK Social Worker Intervention

The Talking about Live Kidney Donation Social Worker Intervention (TALK SWI) was previously developed to improve potential recipients’ pre-emptive activation toward LDKT among patients with chronic kidney disease who had not yet developed kidney failure [20, 22]. TALK SWI is a culturally sensitive theory-based intervention, developed in collaboration with a behavioral psychologist, transplant nephrologists, transplant surgeons, transplant social workers, patient advocacy experts from the National Kidney Foundation of Maryland, and with the input from patients and families with CKD and LDKT experience. It consists of an educational booklet and video coupled with a social worker-led brief behavioral support intervention to help patients and their families overcome barriers to considering and pursuing LDKT. In a recent randomized controlled trial, the TALK SWI was effective in activating patients to consider and pursue LDKT, including their increased engagement in LDKT discussions, their completion of LDKT medical evaluations, and their identification of potential live donors [19]. TALK SWI directly addresses many concerns previously identified among African American patients and families considering LDKT [14] (Table 1).

##### TALK SWI education

The TALK SWI educational component consists of a 20-min video and booklet that are designed for patients and their families to review alone or together prior to a visit with the TALK Social Worker. The video features minority and non-minority patients who have undergone LDKT and their family members discussing their experiences with considering LDKT as a treatment option from the recipient and donor perspectives, as well as health care providers (transplant surgeon, transplant nephrologist, transplant social worker) citing key factors patients and families should consider when contemplating LDKT. It also directly addresses concerns that may pose specific barriers to LDKT for African Americans, including mistrust or fear of the LDKT process, difficulties discussing LDKT, and financial considerations related to pursuing LDKT (Table 2). The educational booklet provides a synopsis of the LDKT process from recipient and donor perspectives. It also includes a listing of publicly available resources from which further information about the LDKT evaluation, transplant, and donation process, clinical risks with LDKT, and financial issues related to LDKT can be obtained. To assist patients and family members with initiating LDKT discussions or addressing complex issues during LDKT discussions (e.g., donor coercion), the booklet also presents several ‘model conversations’ presenting examples on how to initiate and sustain LDKT discussions. The TALK SWI booklet and video were screened to ensure their appropriateness for all persons considering LDKT, including minority and non-minority persons with low (i.e. 4th grade) reading level and low health literacy.

**Table 2:** Barriers to LDKT identified^a^ by patients and family members considering LDKT and addressed by TALK SWI

Patients	Families
Difficulty initiating discussions on own	Feeling overwhelmed by patients’ disease
Concern about being misinterpreted during LDKT discussions	Patients’ denial as barrier to discussions
Concern about burdening family members	Caregiver stress
Concern about guilt/ potential donor coercion	Uncertainty about their own health risks
Financial Concerns	Financial Concerns

^a^Identified in focus groups of African American and non-African American patients with CKD [14]

##### TALK SWI behavioral support

The behavioral support component is a protocol-driven individual and family-based social intervention applying a Social Construction-based Family Problem Solving theoretical framework [23–25]. According to this framework, families are problem solving units whose optimal structure for confronting problems potentially affecting all group members, such as ESRD and LDKT, is achieved when a neutral authority figure is designated as the mediator for relaying messages between all members and encouraging open channels of communication to enable each member to contribute to the problem’s resolution, ultimately enhancing group satisfaction. The protocol specifies that the TALK Social Worker will meet with patient participants considering LDKT during two visits. During the first visit, the Social Worker will meet individually with the patient participant for up to one hour to assess their perceived barriers to completing key behaviors reflecting their consideration and pursuit of LDKT (discussing LDKT with their families, discussing LDKT with their physicians, identifying a potential live donor). The TALK Social Worker employs motivational interviewing techniques to help patients self-identify potential barriers they face toward completing LDKT and to help patients strategize about ways they might overcome barriers. At the time of the first visit, the TALK Social Worker invites patients to bring adult family members and/or friends (henceforth shortened to “family members”) for a second visit. During the second visit, the TALK Social Worker assesses the extent to which previous discussions with family members about patients’ kidney disease had occurred, the results of such conversations, whether family members have communicated about LDKT with patients’ physicians, and any barriers family members perceive toward achieving LDKT [20, 22].

##### Intervention adaptation

We reviewed the original TALK SWI protocol with transplant personnel, including a social worker, at the Duke University Kidney and Pancreas Transplant Program and adapted the TALK SWI into their existing workflows. Adaptation focused on (1) ensuring the intervention adequately addresses patients’ communication and financial needs in social workers’ views, and (2) ensuring the intervention can be feasibly implemented and sustained by transplant centers long-term. For instance, we asked social workers and coordinators to identify the resources they typically provide to patients and families concerned about the financial aspects of LDKT and we incorporated usual work flows regarding financial support into the protocol. Further, in our prior focus groups of patients and families considering LDKT or with LDKT experience, groups identified additional support roles that transplant social workers would be well qualified to fill, including a role as a point of contact for patients and families seeking financial assistance with LDKT [14]. We refined the original TALK SWI behavioral support protocol to include these additional roles that are routinely filled by social workers in transplant centers. We also asked transplant program social workers, nurse coordinators, administrators, and transplant surgeons and nephrologists about clinical workflows entailed with recipient and donor evaluations in an effort to integrate interventions to complement existing clinical programs.

### Live donor financial assistance intervention

We have designed a financial assistance intervention to provide support for potential living kidney donors’ medical and non-medical expenses associated with pursuing live kidney donation. The intervention is modeled after the National Living Donor Assistance Center (NLDAC), a federally funded program administered by the Division of Transplantation (DoT), Healthcare Systems Bureau (HSB), Health Resources and Services Administration (HRSA), United States Health and Human Services (HHS) through a cooperative agreement with the University of Michigan and the American Society of Transplant Surgeons (http://www.livingdonorassistance.org/default.aspx) [21]. It is intended to provide financial support for potential live kidney donors in circumstances where existing federal programs do not provide support. We will offer patient participants the option of enrolling their willing potential live kidney donors (i.e. persons who may wish to pursue live kidney donation) in the financial assistance program, which will provide reimbursement for medical and non-medical expenses related to the evaluation, surgery, and recovery periods associated with live kidney donation. Each patient participant (potential kidney recipient) will be offered a “bank” of $2100.00 from which potential donors can receive reimbursement for live kidney donation related expenses. While multiple people may step forward to be evaluated for donation and incur expenses, the total amount available per patient participant is $2100.00. Our intervention will provide reimbursement to a broad group of potential donors, of which many may not meet NLDAC income requirements. Participants not qualifying for NLDAC may not require the same level of financial reimbursement, or those choosing to forgo NLDAC may desire more broadly applicable financial assistance (e.g., for lost wages from work) than assistance provided by NLDAC (Table 3). Based on national data in 2010, $2100.00 corresponded to 3 weeks of paid leave from work for production or non-supervisory workers [26], approximately 4 weeks of child care, [27] or travel and lodging needs for donors coming from other geographic areas. If effective, our intervention may provide rationale for expanding current qualification requirements for programs such as NLDAC.

**Table 3:** Live donor financial assistance intervention details and qualifying expenses

Feature	Proposed intervention	NLDAC
Financial assistance amount	$2100	$6000
Potential donor and recipient income limits	No	300 % poverty level or less
Proof of donor financial hardship required	No	Yes
Covers travel, hotel, parking and meal costs related to donor evaluation, surgery, and follow-up	Yes	Yes
Covers lost wages from work related to donor evaluation, surgery, and follow-up	Yes	No
Covers child care related costs related to donor evaluation, surgery, and follow-up	Yes	No

We will reimburse potential donors for approved financial assistance through the study by submitting formal original invoices, receipts, and other documentation of their need for reimbursement of qualified medical and non-medical expenses related to live kidney donation evaluation, donation, or convalescence (up to one year after the patient participant enrolls in the study). Qualifying expenses include travel, lodging, meals, incidental expenses (e.g., parking, long-distance phone calls), lost wages, doctors and hospital visits, and childcare costs incurred by the potential donor as part of (1) donor evaluation, clinic visit or hospitalization, (2) hospitalization for the living donor surgical procedure, and/or (3) medical or surgical follow-up clinic visit or hospitalization within one year of the patient participant’s enrollment in the study. Participants will be permitted to utilize the $2100.00 for multiple approved purposes, as long as the total value is not greater than $2100.00. Furthermore, more than one potential donor may draw from these funds, as the total value of reimbursed expenses does not exceed the value of $2100.00 per patient participant. We developed the Live Donor Financial Assistance Intervention following guidelines of the “Organ Donation and Recovery Improvement Act (ODRIA),” (Section 3, 42 U.S.C. 274f) signed into law on April 5, 2004, establishing the authority and legislative parameters to provide reimbursement for travel and subsistence expenses incurred towards living organ donation. The financial assistance program being offered as part of this study is intended to provide reimbursement only in those circumstances when payment cannot reasonably be covered by other sources of reimbursement, including: any State compensation program, an insurance policy, or any Federal or State health benefits program; an entity that provides health services on a prepaid basis; or the recipient of the organ. In order to be reimbursed for travel and qualifying expenses, potential living organ donors must also meet all of the following eligibility criteria and attest to: be a U.S. citizen or U.S. resident; have primary residence in the U.S. or its territories; travel is originating from the donor’s primary residence; donor and recipient attest to full compliance with section 301 of the National Organ Transplant Act (NOTA), as amended (42 U.S.C. 274e) which stipulated in part “…(i)t shall be unlawful for any person to knowingly acquire, receive, or otherwise transfer any human organ for valuable consideration for use in human transplantation if the transfer affects interstate commerce”; the transplant center where the donation procedure occurs attests to its status of good standing with the Organ Procurement and Transplantation Network. Additionally, potential donors are eligible for reimbursement for qualifying expenses incurred toward the intended donation of an organ, even if the donation does not occur.

#### Randomized controlled trial

**Fig. 1:**
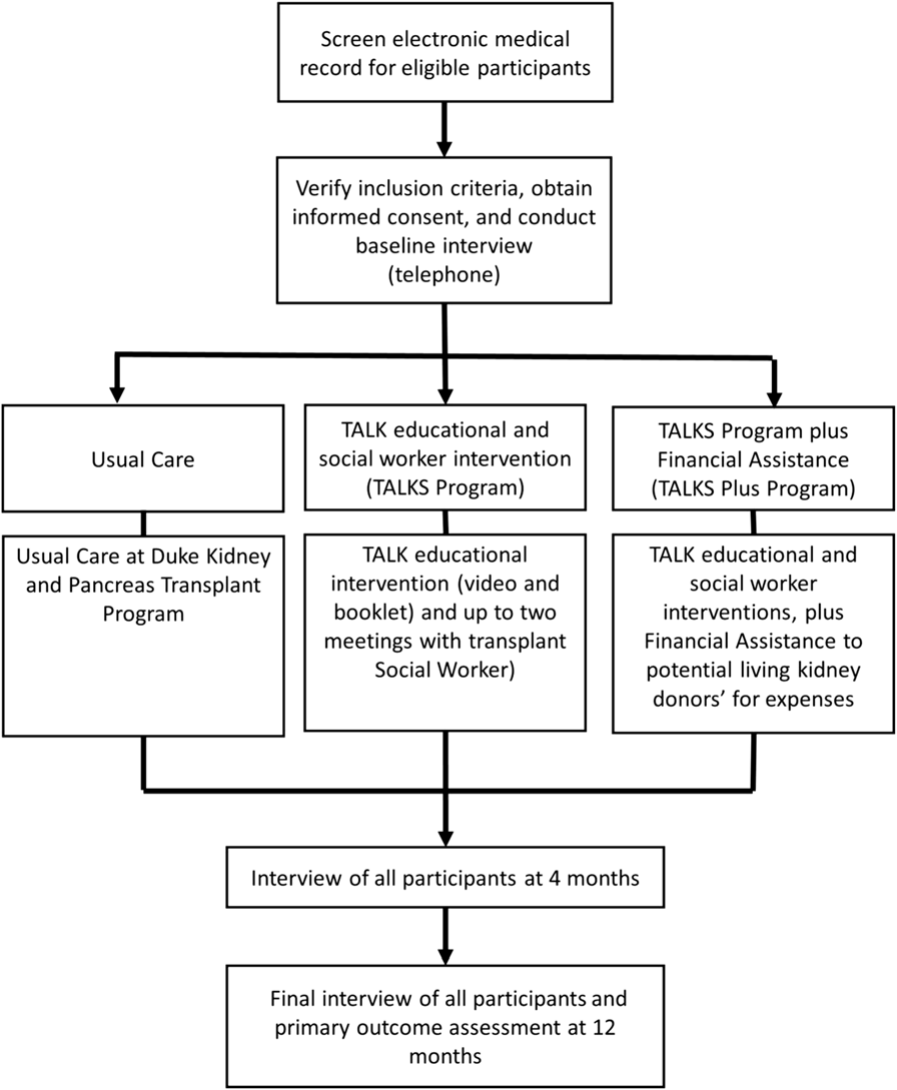
Overview of study design and randomized controlled trial

We will conduct a randomized controlled trial to assess the effectiveness of TALK SWI and the TALK SWI plus financial assistance intervention to improve rates of LDKT among African American potential transplant recipients on the deceased donor kidney waiting list at the Duke Kidney and Pancreas Transplant Program compared to usual care (Fig. 1). We will follow potential recipients for 1 year to assess live kidney donor activation on potential recipients’ behalves (live donor inquiries, completed new live donor evaluations, or live kidney donation) (primary outcome). We will also assess potential recipients’ self-reported behaviors reflecting their pursuit of LDKT, including their conduct of LDKT discussions with physicians and with their families and their identification of potential live donors.

##### Target population

We will target adult African American patients with end stage kidney disease who are listed on the deceased donor kidney waiting list of the Duke Kidney and Pancreas Transplant Program. The Duke Kidney and Pancreas Transplant Program cares for a highly diverse set of patients with end stage kidney disease, with nearly 55 % of the deceased donor kidney transplant waiting list comprised of African Americans annually. African Americans on the deceased donor kidney waiting list at Duke Kidney and Pancreas Transplant Program reflect the local Durham, NC population in which 19 % have incomes below the Federal Poverty Level [28]. The Program currently does not have a social worker program tailored specifically to address communication, logistical or financial barriers to LDKT in African American potential recipients.

##### Screening, eligibility, and recruitment

We will enroll qualifying patients (‘patient participants’) receiving care at the Duke Kidney and Pancreas Transplant Program, their family members and/or friends (‘family member participants’), and their potential donors (‘potential donor participants’) into the study. To identify patient participants, we will screen Duke electronic medical records to identify African American patients who are listed on the deceased donor waiting list at the Duke Kidney and Pancreas Transplant Program with no prior kidney transplant, and who are aged 18 and older. We will obtain appropriate permissions that protect patients’ privacy (i.e., HIPAA waiver) prior to all study procedures. We will mail patients who are potentially eligible to participate a recruitment letter signed by all physicians and surgeons in the Duke Kidney and Pancreas Transplant Program, accompanied by information describing the study. This letter will provide instructions for potential participants to call or email the research team with interest in participating or to opt-out from participation by either returning the provided letter in the self-addressed mailed envelope, or by calling the telephone number provided or emailing study staff prior to the first telephone call. Following a 14-day opt-out period, study staff will contact potential patient participants via telephone to confirm their eligibility for participation in the study, answer any questions, obtain verbal consent, and complete a baseline questionnaire. Random assignment to intervention arms will take place immediately following completion of the baseline questionnaire while consenting patient participants are on the telephone.

Family members or friends of enrolled patient participants (i.e. ‘family member participants’) who attend a social worker visit with patient participants will be asked to provide written consent to participate in the study at the time they present for the social worker visit. Any adult family member or friend who attends will be asked to provide written consent. Potential donor participants interested in the Live Donor Financial Assistance Intervention will only become aware of the intervention through referrals by enrolled patient participants who are randomly assigned to TALK SWI plus financial assistance study arm (see below), who voluntarily choose to make potential donors aware on their own accord, without prompting from study staff or the social worker. Potential donors will contact TALKS Study staff if they are interested in learning more about or want to take part in the TALKS Financial Assistance Intervention. At that time, study staff will address any questions potential donors may have about the intervention. If they decide to participate, study staff will obtain verbal consent over the phone.

#### Random intervention assignment

Using blind and secure allocation by computer, we will randomly assign participants to one of three intervention arms: 1) control group (“Usual Care study arm”); 2) TALK SWI (“TALKS Program study arm”); and 3) TALK SWI plus the Live Donor Financial Assistance Intervention (“TALKS Plus Program study arm”). We will perform outcome and correlate assessments at baseline, 4 months, and 12 months for all patient participants following their enrollment in the study (Fig. 1).

##### Usual care

Patient participants assigned to receive Usual Care will receive care as they usually would through the Duke Kidney and Pancreas Transplant Program. In the Usual Care program, all patients meet with a financial coordinator to review financial programs that may be available to them. They also meet with a social worker to discuss transplantation options, social support, and caregiver identification, and to address any questions patients have about transplantation. Through Usual Care, potential donors have the option of meeting with an external Independent Living Donor Advocate, to address any questions or concerns they have about the donation process.

##### Intervention groups

Study staff will mail the TALK video and booklet to patient participants randomly assigned to the TALKS Program and TALKS Plus Program study arms. A letter accompanying the video and booklet will encourage patient participants to review the 20-min TALK video and booklet and to attend meetings with a study social worker to receive the TALK SWI. The letter will also encourage patient participants to share materials with family members or friends over the age of 18, if they desire. We will invite patient participants assigned to the TALKS Program and TALKS Plus Program study arms to meet with a study social worker for up to an hour to discuss their self-identified barriers to LDKT and strategies to overcome barriers. Following this initial meeting, the social worker will invite patient participants to participate in a second session to discuss possible pursuit of LDKT by themselves or with one or more adult family members or friends. TALK social worker visits occur outside of the usual care setting.

If patient participants are randomly assigned to the TALKS Plus Program study arm, study staff will also mail information about the live donor financial assistance intervention along with the TALK video and booklet. Study staff will review the TALKS Study Financial Assistance Intervention with patient participants assigned to this arm at their first visit with the study social worker.

#### Participant reimbursements

Patient participants will receive $30 for completing each of the questionnaires and $50 for each completed session with the social worker. Family participants attending social worker meetings will receive $20 total for completing the questionnaire and for participating in the social worker session. Potential donors identified by patient participants in the TALKS Plus Program study arm who want to participate in the Live Donor Financial Assistance Intervention will be asked to complete a Live Donor Financial Assistance Intervention Donor Worksheet summarizing expenses as well as sign a Donor Attestation Form to confirm that they understand the legal restrictions of the intervention. Study staff will mail potential donors checks reimbursing approved eligible expenses.

#### Data collection and outcomes

##### Participant characteristics, correlates of LDKT, and social worker meeting content

We will collect information on participant demographics (e.g., age, race, education and income), attitudes/perceptions (e.g., trust in health care team, satisfaction with transplant care), self-reported medical history, and other correlates of LDKT (e.g., health literacy, family function, and self-efficacy with regard to decision-making about LDKT) via telephone questionnaires performed at enrollment, 4 months, and 12 months follow up. In addition to these correlates, we will assess patient participants’ perceptions of the cultural competency of the DKPTP and transplant social workers using the Agency for Health Care Research and Quality CAHPS Cultural Competence Item Set [29], designed to capture patients’ perspectives on the cultural competence of health care providers. Items include information on patient participants’ (a) perceived communication with their transplant health care providers including shared decision-making; (b) experiences of discrimination due to race, ethnicity, insurance, or language; (c) experiences leading to trust or distrust; (d) and linguistic competency (Table 4).

**Table 4:** Study outcomes and assessments

	Baseline	4 months	12 months
Primary outcome: Live kidney donor activation (Composite)			
Live donor kidney transplantation (LDKT)		X	X
Completed live donor evaluations	X	X	X
Live donor inquiries to transplant center	X	X	X
Secondary outcomes			
LDKT discussions with physician	X	X	X
LDKT discussions with family and/or friends	X	X	X
Identification of potential live donor	X	X	X
Belief & knowledge about treatment for kidney failure, interest in LDKT			
Beliefs about treatment for kidney failure	X	X	X
Knowledge of LDKT	X	X	X
Interest in and concerns about LDKT	X	X	X
Knowledge of kidney transplant financial assistance programs	X	X	X
Barriers to and quality of family discussion about LDKT	X	X	X
Mediators and correlates of pursuit of LDKT			
Current treatment information	X	X	X
Socio-demographic Information	X		
Family wealth^a^	X		
Family function (Family APGAR Scale) [32]	X		
Decision self-efficacy about LDKT [33]	X	X	X
Decisional conflict scale [34]	X	X	X
Trust in medical care [35, 36]	X		
Barriers to obtaining information about LDKT		X	X
Depressed mood (PRIME-MD) [37]	X	X	X
Social health (PROMIS-SF) [38, 39]	X	X	X
Risk numeracy [40, 41]	X		
Rapid Estimate of Adult Literacy in Medicine (REALM) [42]	X		
Personal financial well-being scale^a^ [43]	X		
Patient assessment of providers and systems			
Cultural competence of health care providers [29]	X	X	X
In-center dialysis care [44]	X	X	X
Nephrologists’ communication and caring	X	X	X
Fidelity to TALK SWI protocol, intervention uptake and satisfaction			
Participant use of TALK SWI educational materials		X	X
Participant satisfaction with TALK SWI sessions^a^		X	
Transplant social worker adherence to TALK SWI protocol		X	X
Use of financial assistance programs (by donors) and types of expenditures reimbursed		X	X

^a^Information is provided by patient participants and family member participants

In telephone questionnaires administered at 4 months and 12 months follow up, we will ask patient participants randomly assigned to receive TALKS interventions whether they have reviewed the TALK video and booklet and/or shared them with family members. We will also ask patient participants about their perceptions of the video and booklet. We will ask patient and family member participants who attend the social worker meetings their perceptions of the usefulness of these meetings at meeting conclusion via written questionnaire. With participant consent, we will audio record all social worker meetings to assess content and fidelity to the intervention. Social workers will also take notes to document the content of social worker meetings in electronic case report forms. For patient participants randomly assigned to the TALKS Plus Program study arm, we will assess via questionnaire whether patient participants made others (e.g., family members or friends) aware of their participation in the Live Donor Financial Assistance Intervention. For potential donor participants, we will assess the types of expenditures for which they request reimbursement (Table 4).

##### Primary and secondary outcomes

For our primary outcome, we will assess “live kidney donor activation”, defined as the composite rate of live kidney donor inquiries on behalf of patient participants, completed live kidney donor transplant evaluations, and live kidney donor transplants in each arm, ascertained via medical records maintained by the Duke Kidney and Pancreas Transplant Program. Secondarily, we will measure patient participants’ behaviors reflecting their interest and pursuit of LDKT, including: self-reported LDKT discussions with physicians, self-reported LDKT discussions with family, and identification of a potential live donor. We will also record if and when deceased donor transplant occurs.

#### Analysis

Our primary analyses will test whether the TALKS Program is more effective than usual care and whether TALKS plus the Financial Assistance Intervention (TALKS Plus Program) has additional incremental effectiveness compared to the TALKS Program only. We will assess outcomes at 4 months and 12 months after the intervention, enhancing opportunities to ascertain patient participants’ achievement of main outcomes in the event of patient participant attrition or missing data due to missed study visits (e.g., due to hospitalization with ESRD related illness).

The primary outcome is live kidney donor activation (i.e., live donor inquiries to transplantation, completed live donor evaluations, or live donor kidney transplantation). The primary testing contrasts are (1) TALKS Program versus Usual Care, and (2) TALKS Plus Program versus TALKS Program. In secondary analyses, we will test for a trend in effectiveness (TALKS Plus Program more effective than TALKS Program, which is in turn more effective than Usual Care). For the primary testing contrasts, randomly assigned intervention group (i.e., Usual Care, TALKS Program, TALKS Plus Program) will be the main independent variable. For each analysis we will use chi-square statistics to assess the proportion of participants in each group achieving at least one live donor activation outcome over 12 months. In the circumstance that there is imbalance in baseline characteristics between the two groups, we will use multivariable analyses to assess differences in live donor activation (dependent variable) over 12 months accounting for group differences. We will account for multiple comparisons (2 primary comparisons). We will assess the prevalence of missing data and examine robustness of intervention effects under various assumptions regarding plausible patterns of missing data in sensitivity analyses.

#### Sample size estimates

We will enroll and randomly assign 100 patient participants per study arm. Few randomized controlled trials have been previously performed to establish the effectiveness of interventions to improve rates of live kidney donation and LDKT among African Americans. In our own TALK study among chronic kidney disease patients, we identified a 28 % improvement in achievement of LDKT consideration/pursuit behaviors at 6 months. In another randomized controlled trial, home visits to African American families led to a 20 % improvement in live donor inquiries [30]. Based on these studies, we estimate the TALKS interventions will yield a 25 % improvement in live donor activation behaviors. We are not aware of studies directly studying the effect of financial interventions on live donor activation. We expect that potentially willing donors will be enthusiastic about participating in this intervention and estimate at least an additional 20 % incremental increase in live donor activation with the financial assistance intervention. Under usual circumstances, we estimate approximately 25 % of African American patients in the Duke Kidney and Pancreas Transplant Program deceased donor transplant waiting list receive inquiries from live donors interested in being evaluated on waiting list registrants’ behalves each year. Under these assumptions, we estimate we will have approximately 95 % power to detect a 23-30 % difference between TALKS Program study arm and Usual Care study arm at follow up as well as a 21-25 % difference between TALKS Plus Program study arm and the TALKS Program study arm at follow-up. We also estimate we will have 99 % power to observe a trend across the intervention arms, while accounting for multiple comparisons, and accounting for 80 % attrition of participants.

## Discussion

Interventions to improve African Americans’ access to LDKT are sorely needed, as trends indicate few reductions in disparities in receipt of LDKT have been made over the past 10 years [31]. African American patients on the live donor kidney transplant waiting list are likely to be highly motivated to receive transplants and have already overcome some barriers to receiving a transplant by completing their recipient transplant evaluations. Nonetheless, they may not have completed key steps to receiving LDKT, contributing to disparities in their access to this optimal treatment. TALK interventions are designed to be culturally sensitive and to directly target barriers to LDKT previously identified as important to African Americans, but they have not yet been tested among African American patients on the deceased donor kidney waiting list. The Live Donor Financial Assistance Intervention extends TALK interventions by additionally addressing potential donor logistical and financial barriers to LDKT.

To our knowledge, TALKS will be one of the first National Institutes of Health (NIH) funded studies to evaluate the effectiveness of combining educational and behavioral interventions with live donor financial assistance to help African Americans on the waiting list receive LDKT. In addition to providing valuable information on the incremental effectiveness of the live donor financial assistance intervention to improve access to LDKT, information we obtain on the types of expenditures potential donors seek for reimbursement will broaden our understanding of the types of assistance which potential donors need the most. Evidence on the uptake and effectiveness of the financial assistance intervention will help inform current national and regional program resource allocation as well as the need and/or feasibility of expanding current programs.

As one of few clinical trials in the field, the TALKS Study will provide needed rigorous evidence to identify practical and effective strategies to improve disparities in LDKT. If TALKS interventions are effective, their ultimate translation into clinical practice changes will be required to yield substantive and lasting improvements in LDKT rates among African Americans.

## Abbreviations

ESRD: End stage renal disease
LDKT: Live donor kidney transplantation
TALKS: Talking about Living Kidney Donation Support
TALK SWI: Talking about living kidney donation social worker intervention
NLDAC: National Living Donor Assistance Center
DoT: Division of Transplantation
HSB: Healthcare Systems Bureau
HRSA: Health Resources and Services Administration
HHS: Health and Human Services
ODRIA: Organ Donation and Recovery Improvement Act
NOTA: National Organ Transplant Act
